# Supplementary figures and images for: Remodeling of skeletal muscle myosin metabolic states in hibernating mammals
Source: eLife. 2024 May 16;13:RP94616. doi: 10.7554/eLife.94616 (PMC11098559; doi:10.7554/eLife.94616)

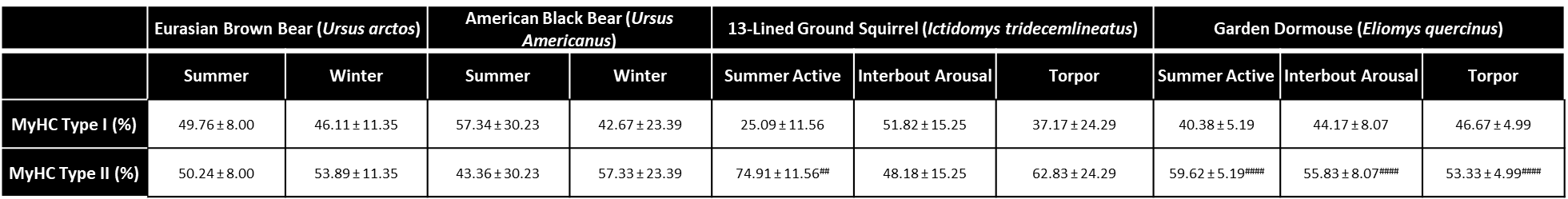

Supplement: Supplementary file 1. — Table demonstrating the percentage of fibers which were analyzed during Mant-ATP chase assays that were either MyHC type I or MyHC type II. Data is presented as mean for each animal ± SD. One-way ANOVA was used to calculate significance between hibernation periods in I. tridecemlineatus and E. quercinus. Student’s t-test was used to calculate significant between hibernating periods in U. arctos and U. americanus and between MyHC type I and MyHC type II in all animals. ##=p < 0.01 vs MyHC type I in corresponding group. ####=p < 0.0001 vs MyHC type I in corresponding group. n=5 individual animals per group. [file elife-94616-supp1.docx]

A


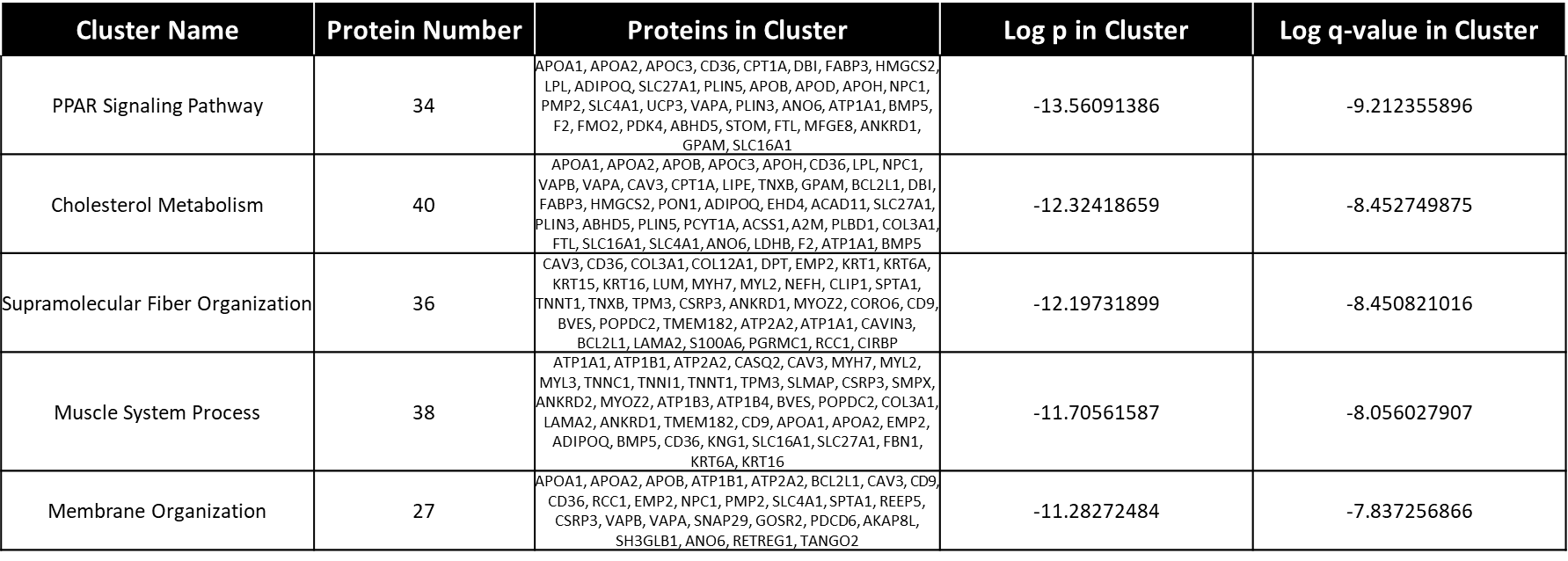


B


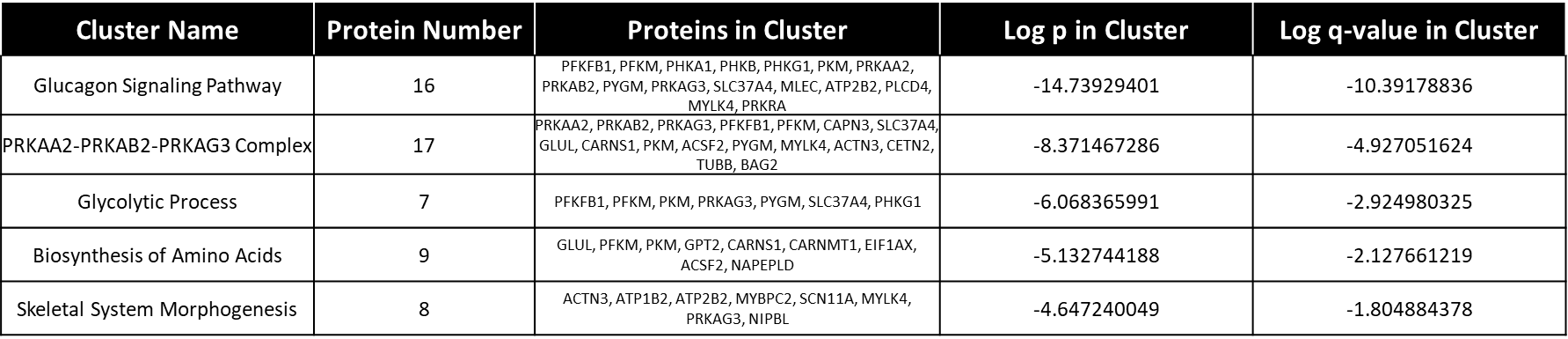

Supplement: Supplementary file 2. — A. Table details the list of proteins which were found to be differentially upregulated in torpor vs SA in each corresponding ontological cluster as identified by Metascape. Clusters are arranged by order of statistical significance. Proteins are listed in alphabetical order within each cluster. B. Table details the list of proteins which were found to be differentially downregulated in torpor vs SA in each corresponding ontological cluster as identified by Metascape. Clusters are arranged by order of statistical significance. Proteins are listed in alphabetical order within each cluster. n=5 individual animals per group. [file elife-94616-supp2.docx]

A


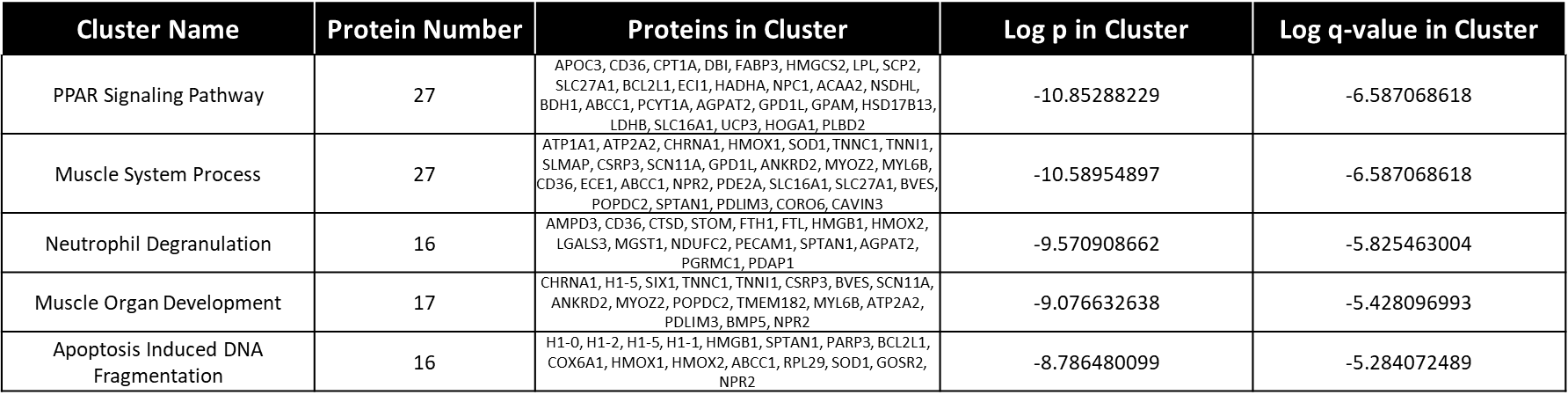


B


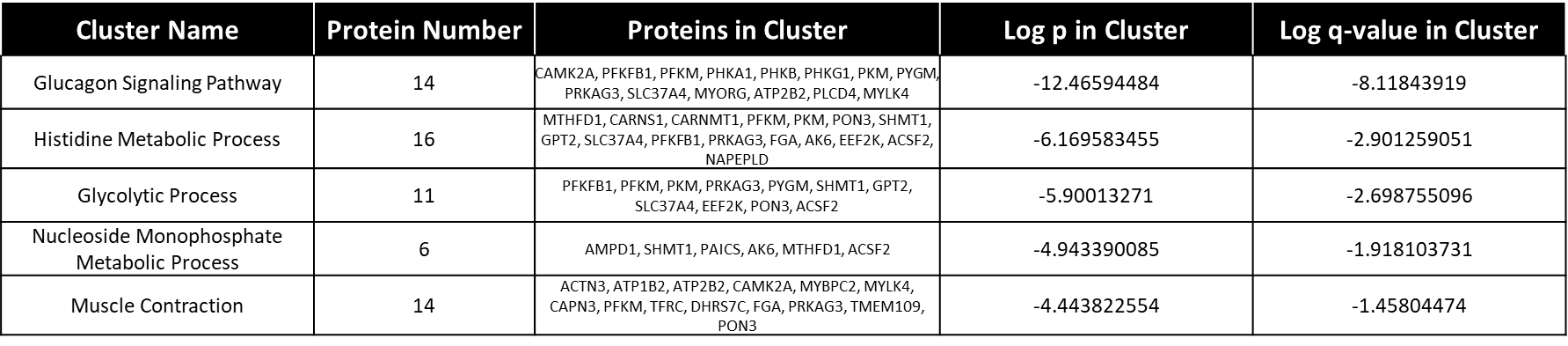

Supplement: Supplementary file 3. — A. Table details the list of proteins which were found to be differentially upregulated in IBA vs SA in each corresponding ontological cluster as identified by Metascape. Clusters are arranged by order of statistical significance. Proteins are listed in alphabetical order within each cluster. B. Table details the list of proteins which were found to be differentially downregulated in IBA vs SA in each corresponding ontological cluster as identified by Metascape. Clusters are arranged by order of statistical significance. Proteins are listed in alphabetical order within each cluster. n=5 individual animals per group. [file elife-94616-supp3.docx]

A


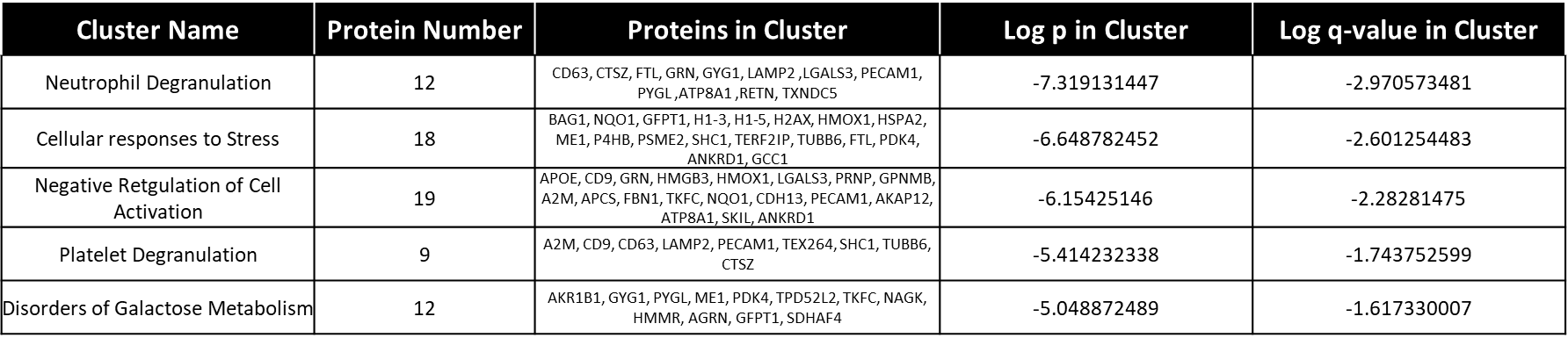


B


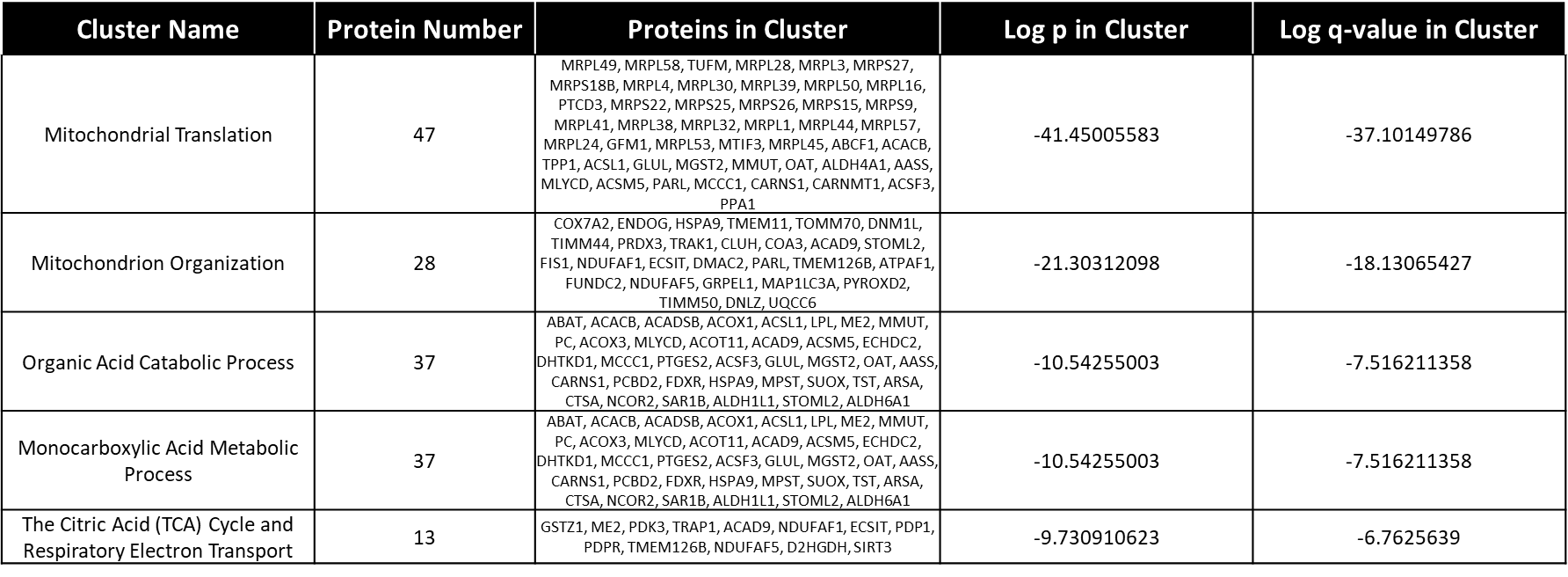

Supplement: Supplementary file 4. — A. Table details the list of proteins which were found to be differentially upregulated in winter vs summer in each corresponding ontological cluster as identified by Metascape. Clusters are arranged by order of statistical significance. Proteins are listed in alphabetical order within each cluster. B. Table details the list of proteins which were found to be differentially downregulated in winter vs summer in each corresponding ontological cluster as identified by Metascape. Clusters are arranged by order of statistical significance. Proteins are listed in alphabetical order within each cluster. n=5 individual animals per group. [file elife-94616-supp4.docx]
